# Medication administration error: magnitude and associated factors among nurses in Ethiopia

**DOI:** 10.1186/s12912-015-0099-1

**Published:** 2015-10-21

**Authors:** Senafikish Amsalu Feleke, Muluadam Abebe Mulatu, Yeshaneh Seyoum Yesmaw

**Affiliations:** Department of Reproductive Health, Institute of Public Health, University of Gondar, Gondar, Ethiopia; Internal Medicine Department, Felege Hiwot Referal Hospital, Bahir Dar, Ethiopia; Department of Nursing, Bahir Dar University, Bahir Dar, Ethiopia

**Keywords:** Medication errors, Magnitude, Nurses, Developing country

## Abstract

**Background:**

The significant impact of medication administration errors affect patients in terms of morbidity, mortality, adverse drug events, and increased length of hospital stay. It also increases costs for clinicians and healthcare systems. Due to this, assessing the magnitude and associated factors of medication administration error has a significant contribution for improving the quality of patient care. The aim of this study was to assess the magnitude and associated factors of medication administration errors among nurses at the Felege Hiwot Referral Hospital inpatient department.

**Methods:**

A prospective, observation-based, cross-sectional study was conducted from March 24–April 7, 2014 at the Felege Hiwot Referral Hospital inpatient department. A total of 82 nurses were interviewed using a pre-tested structured questionnaire, and observed while administering 360 medications by using a checklist supplemented with a review of medication charts. Data were analyzed by using SPSS version 20 software package and logistic regression was done to identify possible factors associated with medication administration error.

**Result:**

The incidence of medication administration error was 199 (56.4 %). The majority (87.5 %) of the medications have documentation error, followed by technique error 263 (73.1 %) and time error 193 (53.6 %). Variables which were significantly associated with medication administration error include nurses between the ages of 18–25 years [Adjusted Odds Ratio (AOR) = 2.9, 95 % CI (1.65,6.38)], 26–30 years [AOR = 2.3, 95 % CI (1.55, 7.26)] and 31–40 years [AOR = 2.1, 95 % CI (1.07, 4.12)], work experience of less than or equal to 10 years [AOR = 1.7, 95 % CI (1.33, 4.99)], nurse to patient ratio of 7–10 [AOR = 1.6, 95 % CI (1.44, 3.19)] and greater than 10 [AOR = 1.5, 95 % CI (1.38, 3.89)], interruption of the respondent at the time of medication administration [AOR = 1.5, 95 % CI (1.14, 3.21)], night shift of medication administration [AOR = 3.1, 95 % CI (1.38, 9.66)] and age of the patients with less than 18 years [AOR = 2.3, 95 % CI (1.17, 4.62)].

**Conclusion:**

In general, medication errors at the administration phase were highly prevalent in Felege Hiwot Referral Hospital. Documentation error is the most dominant type of error observed during the study. Increasing nurses’ staffing levels, minimizing distraction and interruptions during medication administration by using no interruptions zones and “No-Talk” signage are recommended to overcome medication administration errors. Retaining experienced nurses from leaving to train and supervise inexperienced nurses with the focus on medication safety, in addition providing convenient sleep hours for nurses would be helpful in ensuring that medication errors don’t occur as frequently as observed in this study.

**Electronic supplementary material:**

The online version of this article (doi:10.1186/s12912-015-0099-1) contains supplementary material, which is available to authorized users.

## Background

Medication error (ME) is broadly defined as any error in the prescribing, dispensing, or administration of a drug. ME is the single most preventable cause of patient harm [1]. Medication administration error (MAE) is defined as “any difference between what the patient received or was supposed to receive and what the prescriber intended in the original order” [2]. MAE is one of the risk areas of nursing practice and occurs when a discrepancy occurs between the drug received by the patient and the drug therapy intended by the prescriber [3].

As the global denominator, the distributional epidemiology of MAE showed that, the majority of these errors involved either dose omissions (42 %) or wrong time administration (50 %) [4]. The National Patient Safety Agency of UK revealed that, MAE is common and this occurs in 50 % of all drug medication administrations in hospitals [5]. In the USA, MAE occurs in 5 to 20 % of all drug administrations, costing the healthcare system an extra $380 million and estimated to harm at least 1.5 million patients per year, with about 400,000 preventable adverse events [6]. MAE in East Africa is common and the error rate ranges from 9.4 to 80 % of all medication administrations [7]. The prevalence of MAE in Jimma, Ethiopia within the intensive care unit (ICU) and pediatric ward showed 51.8 and 90.8 %, respectively [8, 9].

MAE is one of the most common types of adverse events for hospital admitted patients, and the most frequent cause of preventable death [10]. 38 % of MAE are serious or fatal, and 42 % of those are preventable [10]. MAE has a significant impact on patients in terms of morbidity, mortality, adverse drug event, and increased length of hospital stay. In addition, it increases costs to clinicians and healthcare systems [11]. In the UK, 26 % of MAE were potentially serious, with fatal events which led patients to aspiration pneumonia and intracranial hemorrhage [12]. In Germany, 70 % of all intravenous medications administered had at least one clinical error, and a quarter of these were serious errors likely to result in permanent harm to patients [13].

The most common factors which contribute to MAE are failure to check the patient’s identification prior to administration, the storage of similar preparations in similar areas, and environmental factors like nurse interruptions whilst undertaking a drug round [3]. Other factors which contribute to MAE are characteristics of the nurse (age, sex, years of experience, year in the specific unit, nurse-to-patient ratio and educational status), route, and time of drug administration [14]. Inaccurate documentation and poor communication during change of shifts in the hospitals also contribute to MAE [15, 16].

Bar-coded medication administration (BCMA) and ensuring the patent identifications and following medication rights can help reduce medication administration error by 54–87 % [6]. Medication administration errors can also be prevented by voluntary reporting, direct observation, manual chart review, and computerized techniques [17]. Training and competency of the nurse, medication administration policies, continuous quality improvement efforts of the nurse, clear and accurate documentation, patient education, and teamwork help decrease medication administration error in hospitals [18].

Therefore, assessing the magnitude and associated factors of MAE will produce information that can be used by program managers and stakeholders in the planning and interventions of medication administration errors. Detecting the problem also helps to take corrective measurement for improving medication safety and quality, patient outcomes, adverse drug events, and quality of hospital services.

## Methods

### Study design and set up

An institutional-based, cross-sectional study was conducted in Bahir Dar, Ethiopia, at Felege Hiwot Referral Hospital (FHRH) from March 24–April 7, 2014. The STROBE guidelines were used to ensure the reporting of this cross-sectional study. The filled STROBE checklist is added as in Additional file 1. Nursing encompasses autonomous and collaborative care of individuals of all ages, families, groups and communities, sick or well, and in all settings. Nursing includes the promotion of health, prevention of illness, and the care of ill, disabled and dying people. Advocacy, promotion of a safe environment, research, participation in shaping health policy, in-patient and health systems management, and education are also key nursing roles.

In Ethiopia, strengthening the nursing workforce is central to strengthening health systems. Currently the NEPI project, support for establishment of regulatory body for nurses and midwives in Ethiopia, plays a critical role in strengthening health systems through the development of nursing and midwifery platform, increasing the quantity, quality and relevance of nurses, improving their retention and distribution, and strengthening nursing leadership.

According to the Ethiopian Ministry of Health and Education, there are different educational paths to becoming a nurse: a diploma from an accredited nursing program or hospital, or a bachelor’s degree (Bachelor of Science in Nursing). Diploma tracks have become less popular over the years, as most candidates opt for bachelor’s degrees, due to their availability and versatility. Moreover, nurses can be generalists or obtain Master’s degrees and additional certifications to specialize in a specific area such as pediatric, surgical or just about any medical specialty. Although there are many different types of nursing careers, each with a different set of responsibilities, there is one primary consistency among all nurses of any type, which is the “nursing process”, according to the ANA. The nursing process outlines how a nurse approaches each patient encounter, and includes five steps: assessment, diagnosis, planning, implementation, and evaluation. As with many careers in the healthcare industry, nursing offers very high job stability, as well as a wide variety of options in terms of schedules, locations, and levels of responsibility. Also, many nurses like the rewarding nature of nursing work, which allows them to truly impact the lives of others who are most in need of assistance. Also, nursing can be very lucrative especially if you move into more specialized roles or move up into leadership and management roles.

### Sample size and sampling procedure

Sampling was done using non-probability convenience technique, in which the sum of all the medications which were administered by 85 nurses was taken as the sample size. Therefore, all medication administration interventions to all patients in the inpatient departments by 85 nurses during the data collection period with different medication administration schedule were included.

### Data collection tool and procedure

Data were collected and supervised by eight BSc. nurses and two MSc. nurses respectively. Before the actual work, data collectors and supervisors were given intensive training for two days about the objective of the study, the format of the questionnaire and checklist, procedures of observation and methods of reporting to supervisors and principal investigators. After the training, the questionnaire and checklist were pretested on five nurses who were working in Debre Markose Referral Hospital. Data on medication administration were collected through face-to-face interviews by using a structured questionnaire and by directly observing using checklist. In addition to this, immediately after observation, data on recorded observation were compared with the physicians order by referencing the patient’s chart. The content of the data collection formats were design to record the nurse’s demographics and work experience, patient’s demographics and all data regarding the patient’s medication intervention, date and time that specific drug was prescribed and administered, including the name of the drug, dosage, dosage given, frequency, and route of medication administration. The English version of the questioner is added in Additional file 2.

The appropriateness of the instrument was measured through a pre-testing exercise, and the constraining factors were rectified. Prior to applying the survey instrument, the researchers engaged different expert reviewers as subject matter specialists at Gondar University Hospital to evaluate and finalize the instrument. Regarding the reliability, the study used Cronbach’s coefficient alpha to measure consistency, complementarily and correlation coefficient. To generate the Cronbach’s alpha results, validation of the instrument was conducted through a pilot study and the results obtained had an overall Cronbach’s alpha of (r) = 0.72.

The following operational definitions were used: Medication administration error: A medication error (time, dose, missed drug, unauthorized, route, technique, and documentation errors) that occurs while the time of administering IV, IM, SC, and PO medication to the patient by the nurse. Missed drug error: Failures to administer a prescribed medication while the drug available at the patient bedside. Unauthorized drug error: Medication administered was not authorized by the prescriber. Technique error: The nurse performs less than 50 % among the procedure put at the technique competency checklist for medication administration. Wrong dose error: Medication dose or quantity different from that of prescribed. Wrong time error: There is greater or less than 30 min difference between the ordered time and the time in which the medication is administered. Wrong route error: When there is a difference between the ordered routes of medication administration with the actual route of administration. Documentation error: Medication that is administered to the patient but not documented in medication administration record sheet.

### Data processing and analysis

Data were checked for completeness and entered into EPI INFO version 3.5.3 statistical software and then exported to SPSS version 20 for further analysis. Multiple logistic regression was used to identify variables independently associated with MAE. The strength of association was interpreted using the adjusted odds ratio with 95 % CI. The criterion for statistical significance was set at a *p* value of 0.05.

### Ethical considerations

Ethical clearance was obtained from University of Gondar through the Ethical Committee of the Department of Nursing. Communication with the Amhara Health Bureau and FHRH was made through a formal letter obtained from the Department of Nursing. After the purpose and objective of the study had informed, verbal consent was obtained from the nurses and patients who were included in the study. Verbal consent was obtained because of two reasons: the first is that the topic was not considered sensitive enough to require written consent, and the second is that written consent can act as a deterrent for participants with political concerns. Participants were also informed that participation was on a voluntary basis and that they have the right to withdraw at any time if they are not comfortable with the study. In order to keep confidentiality, all data were kept anonymously in the observational checklist and interview questionnaire.

## Results

### Socio-demographic characteristics of the study participants

Out of 85 study samples, 82 nurses were interviewed and observed making the response rate of 96.5 %. Majority (84 %) of them were female. The mean age of the respondents was 31.13 years with SD of 6.4 years. Majority (85.4 %) of the respondents was diploma nurse and most (65.9 %) of them had working experiences of less than 10 years (Table 1).

**Table 1:** Percentage Distribution of the study participants by Socio Demographic Characteristics, Bahir Dar FHRH inpatient departments, Northwest Ethiopia, March 2014

Variables	Frequency	Percentage	Mean and SD
	(*n* = 82)	(100 %)	
Sex			
Male	13	15.9	
Female	69	84.1	
Age in year			
18–25	17	20.7	
26–30	36	43.9	31 ± 6.4
31–40	18	22.0	
> 40	11	13.4	
Educational status			
Diploma	70	85.4	
BSc	12	14.6	
Religion			
Orthodox	76	92.7	
Muslim	3	3.7	
Protestant	3	3.7	
Working experience in year			
≤ 10	54	65.9	
> 10	28	34.1	

### Characteristics of patients who were included during the observation

A total of 263 patients were included while the nurse administered their medication. Out of 263 patients, more than half (53.6 %) of them were female and majority (82.2 %) of them were aged18 and above. Regarding their admission room, majority (69) of them were admitted to the surgical ward, followed by the medical ward (59) and pediatric ward (47).

### Characteristic of the observed drugs

A total of 360 medication administrations were observed at the selected wards of the FHRH inpatient department. More than three fourths (77.8 %) of the medications were antibiotics. Concerning shift and unit of observation, 185 (51 %) of the medications were observed during working time from Monday–Friday and 87 (24.2 %) were observed in medical ward. In regards to route of administration, the majority of the observed drugs (89.4 %) were IV injection followed by IM injection (7.8 %).

### Number of medication administration error related to observed patients

Among 263 patients observed during the study, 260 (98.8 %) of them had faced at least one type of medication administration error and more than half (56.7 %) of them had faced greater than or equal to three types of medication administration errors.

### Magnitude of medication administration errors

**Fig. 1:**
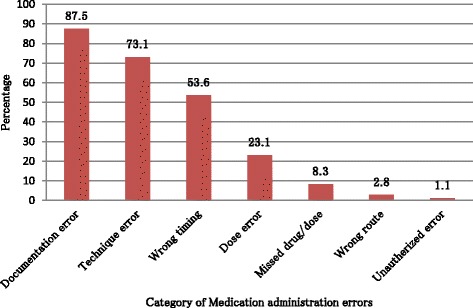
Medication administration error categories in inpatient department of FHRH Bahir Dar, Northwest Ethiopia, March 2014

Out of 360 medication administration interventions, the majority (98.1 %) of medications had at least one type of medication administration error. Among this, 42 (11.9 %) had only one type medication administration error, 112 (31.7 %) had two type of errors, 168 (47.6 %) had three types of errors and the rest 31 (8.8 %) had more than three types of medication administration error. From the total medication administration intervention, more than half (56.4 %) were labeled as medication administration errors. From 87 medication administration interventions which were given at medical unit, 42 (47.2 %) had medication administration error and also in emergency ward from 12 observed medications, all of it had medication administration error. Documentation error, technique error and wrong timing contributed for 315 (87.5 %), 263 (73.1 %), and 193 (53.6 %) of the medication administration errors, respectively (Fig. 1). Types of MAE and some observed examples during the study period are listed in (Table 2).

**Table 2:** Types of medication administration error and some observed examples in the inpatient departments of Bahir Dar FHRH, March 2014

Type of MAEs	Some observed examples
Technique error	Mostly during administration, the nurses used only one glove for different patients and they are not changed the glove even if it has visible contamination.
	Most of the nurses did not wash their hands before medication administration.
	Some of the nurses had not used safe wastage disposal system like they remain the injectable syringe and vial container at the patient bed.
Wrong route	The observer observed while Insuline given intradermally instead of subcutaneous route.
Missed drug (doss)	Quinine mostly was run for more than 8 h instead of 4 h and the 2nd dose missed.
	Methrindazole IV medication mostly missed at 2 PM.
Time error	Most 6 PM medications were being given at 4:30 PM.
Documentation error	Most of the nurses did not document after administration of the drug.
	If the nurses documented before administering the medications, they did not cancelled what they documented even if the patient refused or can’t afford to buy the drug.
Dose error	Instead of administering 2 g of Ceftriaxone, the nurse administered 1 g.
	Usually at pediatric ward, the nurse did not calculate the exact doses of medication.
Unauthorized drug error	Instead of IV Ciprofloxaciline, IV Ceftriaxone was administered. The observer observed a nurse while she was giving Quinine IV instead of Plasil IV.

### Factors associated with medication administration error

Based on the bivariate analysis, the factors found to be significantly associated with medication administration error were age of the respondent and the patient, nurse’s working experience, interruption of the nurses during time of medication administration, shift of medication administration, nurse to patient ratio and route of medication administration.

Out of variables which were entered to multiple logistic regression, age of the respondents and the patient, nurse’s working experience, interruption of the nurses at the time of medication administration, shift of medication administration, and nurse to patient ratio were found to be significantly associated with medication administration error at *p*-value of < = 0.05.

Nurses with the age group of 18–25 years and 26–30 years were 3 times [AOR = 2.9, 95 % CI (1.65, 6.38)] and 2 times [AOR = 2.3, 95 % CI (1.55, 7.26)] more likely to make medication administration error respectively as compared to those with the age greater than 40 year. In addition, nurses with the age group of 31–40 years were 2 times [AOR = 2.1, 95 % CI (1.07, 4.12)] more likely to make medication administration error respectively as compared to those with the age greater than 40 year.

Nurses who had work experience of less than or equal to 10 years were 2 times [AOR = 1.7, 95 % CI (1.33, 4.99)] more likely to make an error when compared to those who had experience greater than 10 years.

Nurse to patient ratio was also found to be one of the strong predictors of MAE. Respondents who had nurse to patient ratio of 7–10 and greater than 10 were 2 times [AOR = 1.6, 95 % CI (1.44, 3.19)] and 2 times [AOR = 1.5, 95 % CI (1.38, 3.89)] more likely to make an error respectively when compared to nurse to patient ratio of 1–6. Nurses who face interruption during medication administration were 2 times [AOR = 1.5, 95 % CI (1.14, 3.21)] more likely to make medication administration error as compared to those who administered without interruption.

In addition, nurses who were administering medication at night were 3 times [AOR = 3.1, 95 % CI (1.38, 9.66)] more likely to made medication administration error when compared to those who were administering medication during the day.

Patients less than 18 years of age were 2 times [AOR = 2.3, 95 % CI (1.17, 4.62)] more likely to face medication administration error than as compared to those with the age greater than or equal to 18 years (Table 3).

**Table 3:** Bivariate and Multivariate analysis of factors associated with medication administration error among nurses at FHRH inpatient department, Bahir Dar, Northwest Ethiopia, March 2014 (*n* = 360)

Variables	Medication administration error		OR with 95 % CI	
	Yes	No	Unadjusted	Adjusted
Nurse’s age in year				
18–25	52	29	2.6(1.29,5.02)	2.9(1.65,6.38)
26–30	83	48	2.5(1.33,4.55)	2.3(1.55, 7.26)
31–40	49	36	1.9(1.02,3.75)	2.1(1.07,4.12)
> 40	26	37	1	1
Educational status				
Diploma	183	130	1.1(0.56,1.94)	^a^
BSC	27	20	1	
Working experience in years				
≤ 10	146	89	1.6(1.24,4.88)	1.7(1.33,4.99)
> 10	64	61	1	1
Duration in the specific unit in month				
< 3	32	30	0.7(0.37,1.27)	^a^
3–6	106	74	0.9(0.57,1.43)	
> 6	72	46	1	
Nurse to patient ratio				
1–6	57	52	1	1
7–10	93	56	1.5(1.39,3.88)	1.6(1.44,3.19)
> 10	60	42	1.3(1.08,4.89)	1.5(1.38,3.89)
Interruption during medication administration				
Yes	132	83	1.4(1.09,3.09)	1.5(1.14,3.21)
No	78	67	1	1
Time of medication administration				
6 AM	62	46	2.3(1.01,5.22)	^a^
12 AM	18	18	1.7(0.55,3.52)	
2 PM	29	29	1.7(0.98,5.57)	
6 PM	71	32	3.7(0.57,4.01)	
10 PM	10	13	1.3(0.67,2.45)	
12 PM	12	20	1	
Shift of medication administration				
Night	126	51	2.9(1.19,7.10)	3.1(1.38,9.66)
Working time	84	99	1	1
Patient age in year				
< 18	50	22	1.6(1.05,3.16)	2.3 (1.17,4.62)
≥ 18	168	120	1	1
Route of administration				
IV	197	126	2.9(1.42,5.88)	^a^
Others (IM, PO and SC)	13	24	1	

^a^Not significant in the multivariate analysis (back ward stepwise logistic regression)

## Discussion

Medication errors have been identified as the most common type of error affecting the safety of patients and the most common single preventable cause of adverse events. MAEs are most often made by nurses administering medications on the patient care unit [19]. This study found that the magnitude of MAEs was 56.4 %, and documentation error followed by technique error was the most common types of medication administration errors. Nurse’s working experience, interruption of the nurses at the time of medication administration, shift of medication administration, and nurse to patient ratio were found to be significantly associated with medication administration error at *p*-value of < = 0.05.

This study revealed that the magnitude of medication administration error was 56.4 %; this finding was higher than those of studies conducted in the Netherland (21.2 %), Paris teaching hospital (27.6 %), Paris Pediatric unit (31.3 %) and Morocco ICU (15.5 %) [20–23]. The possible reason for the difference could be due to a difference in the number of researched clinical units; in which the above researches were done only by involving a single clinical unit, but this research involved different clinical units. In addition, unlike our set up, the above studies were done in a developed country in which computerized recording system, double checking of dangerous medications, voluntary error reporting, and follow up were performed.

Also this finding is higher than that of a study conducted in Jimma University Hospital ICU which was 51.8 % [8]. The possible explanation can be that the Intensive care unit may be given special consideration by hospital staff, and the nurse to patient ratio may be lower compared to the other units.

This finding is lower than the study conducted in the Jimma University Hospital Pediatric unit, which was 89.9 %, and in India Pediatric unit, 68.7 % [9, 24]. Our study involved both Pediatric and Adult units, unlike the Jimma and Indian studies which were conducted only in Pediatric wards, where frequent errors of medication administration are performed.

In this study, a total of 360 medication administrations were observed. From those, 98.1 % of the medication experienced at least one medication administration error. This finding is in line with research done in Sydney, Australia [25].

A substantial number of studies have identified that age of the respondent and working experience were significantly associated with medication administration error [25–27]. This study also supported the above claim. This can be explained by the fact that medication administration is one of the nurse’s practices that improves with age and experience. In addition, nurses with more years and work experience have greater knowledge and skills related to medication administration. They are also very familiar with different types of medications.

This finding states that the number of patients under the nurse is significantly associated with medication administration error. This is in agreement with a study conducted in Paris [21]. This might be due to the fact that, in addition to medication administration, the nurses have a number of duties for the admitted patients in the hospital. So when the number of patients under the nurse increases, he/she is exposed to work over-load which results in MAEs.

Interruption of the nurse at the time of medication administration also contributes to MAE. This finding is consistent with the study done in Sydney, Australia [28]. This can be explained by the fact that, since medication preparation and administration need concentration, interruptions during these activities leads to cognitive failures among nurses in relation to working memory and attentiveness.

Nurses need to be alert enough to provide safe care for their patients and alert enough to detect and correct the errors. Nurses who work night shifts can experience circadian disturbance resulting in disturbed sleep exhaustion, and performance impairment. Our study corroborates this statement: medications administered at night were 2 times more likely to have MAE than when compare to those administered during the day.

The age of the patient was significantly associated with medication administration error. Patient age <18 years were 2 times more likely to face medication administration error as compared to those adults age greater than or equal to 18 years. This might be associated with the level of noise in the pediatric ward compared to the adult ward: the nurses may face difficulties in concentration. Also, medication dosage for pediatric patients need more mathematical calculations, which can lead the nurses to encounter different types of MAEs.

Generalization of the findings presented should be made with caution because of the following limitations: small sample size and lack of random variation in a study's estimates were the main limitations of the study.

## Conclusions

In conclusion, medication errors at the administration phase were highly prevalent in FHRH. Each medication and each patient had at least one type of medication administration error. Documentation error was the most dominant type of error followed by technique and time error respectively. Organizational factors such as error reporting systems and routine checks could possibly help in handling the problem of medication errors. Also, increasing nurse’s staffing levels, minimizing distraction and interruptions during medication administration by decreasing overcrowding and by using no interruptions zones and “No-Talk” signage are recommended to overcome MAEs. Hospital managers should strive to retain experienced nurses from leaving and train and supervise inexperienced nurses with a focus on medication safety. In addition, an administration control to provide convenient sleep hours will help nurses in improving sleep circadian rhythms and reduce MAEs.

## Additional files

Additional file 1:
**STROBE Checklist for Medication Administration Error: Magnitude and Associated factors among Nurses in Ethiopia.** (PDF 187 kb) Additional file 2:
**English version questioner for Medication Administration Error: Magnitude and Associated factors among Nurses in Ethiopia.** (PDF 305 kb)

## Abbreviations

AOR: Adjusted odds ratio
BCMA: Bar-coded medication administration
BSc: Bachelor of science
CI: Confidence interval
COR: Crude odds ratio
FHRH: Felege Hiwot Referral Hospital
ICU: Intensive care unit
IM: Intramuscular
IV: Intravenous
MAE: Medication administration error
ME: Medication error
MSC: Master of science
PO: Per Os
SC: Subcutaneous
SD: Standard deviation
