# Supplementary material for: Medication administration error: magnitude and associated factors among nurses in Ethiopia
Source: BMC Nurs. 2015 Oct 21;14:53. doi: 10.1186/s12912-015-0099-1 (PMC4618536; doi:10.1186/s12912-015-0099-1)
Supplement: Additional file 1: — STROBE Checklist for Medication Administration Error: Magnitude and Associated factors among Nurses in Ethiopia. (PDF 187 kb) [file 12912_2015_99_MOESM1_ESM.pdf]

STROBE Statement—Checklist of items that should be included in reports of ***cross-sectional studies***

	Item No	Recommendation
**Title and abstract**	1	(*a*) Medication administration error: Magnitude and associated factors among nurses in Ethiopia
		(*b*) A prospective, observation-based, cross-sectional study was conducted from March 24 - April 7, 2014 at the Felege Hiwot Referral Hospital inpatient department. The aim of this study was to assess the magnitude and associated factors of medication administration errors among nurses at the Felege Hiwot Referral Hospital inpatient department. A total of 82 nurses were interviewed using a pre-tested structured questionnaire, and observed while administering 360 medications by using a checklist supplemented with a review of medication charts. Data were analyzed by using SPSS version 20 software package and logistic regression was done to identify possible factors associated with medication administration error.<br>The incidence of medication administration error was 199(56.4%). The majority (87.5%) of the medications have documentation error, followed by technique error 263(73.1%) and time error 193(53.6%). Variables which were significantly associated with medication administration error include nurses between the ages of 18-25 years [Adjusted Odds Ratio (AOR)=2.9, 95% CI (1.65,6.38)], 26-30 years [AOR=2.3, 95% CI (1.55, 7.26)] and 31-40 years [AOR=2.1, 95% CI (1.07, 4.12)], work experience of less than or equal to 10 years [AOR=1.7, 95% CI (1.33, 4.99)], nurse to patient ratio of 7-10 [AOR=1.6, 95% CI (1.44, 3.19)] and greater than 10 [AOR=1.5, 95% CI (1.38, 3.89)],interruption of the respondent at the time of medication administration[AOR=1.5, 95% CI (1.14, 3.21)],night shift of medication administration [AOR=3.1, 95% CI (1.38, 9.66)]and age of the patients with less than 18 years [AOR=2.3, 95% CI (1.17, 4.62)].<br>In general, medication errors at the administration phase were highly prevalent in Felege Hiwot Referral Hospital. Documentation error is the most dominant type of error observed during the study. Increasing nurses' staffing levels, minimizing distraction and interruptions during medication administration by using no interruptions zones and "No-Talk" signage are recommended to overcome medication administration errors. Retaining experienced nurses from leaving to train and supervise inexperienced nurses with the focus on medication safety, in addition providing convenient sleep hours for nurses would be helpful in ensuring that medication errors don't occur as frequently as observed in this study.
**Introduction**		
Background/rationale	2	Medication error (ME) is broadly defined as any error in the prescribing, dispensing, or administration of a drug. ME is the single most preventable cause of patient harm [1]. Medication administration error (MAE) is defined as "any difference between what the patient received or was supposed to receive and what the prescriber intended in the original order"[2]. MAE is one of the risk areas of nursing practice and occurs when a discrepancy occurs between the drug received by the patient and the drug therapy intended by the prescriber [3].<br>As the global denominator, the distributional epidemiology of MAE showed that, the majority of these errors involved either dose omissions (42%) or wrong time administration (50%) [4]. The National Patient Safety Agency of UK revealed that, MAE is common and this occurs in 50% of all drug medication administrations in hospitals [5]. In the USA, MAE occurs in 5 to 20% of all drug administrations, costing the healthcare system an extra $380 million and estimated to harm at least 1.5 million patients per year, with about 400,000 preventable adverse events [6]. MAE in East Africa is common and the error rate ranges from 9.4 % to 80 % of all medication administrations [7].

		The prevalence of MAE in Jimma, Ethiopia within the intensive care unit (ICU) and pediatric ward showed 51.8% and 90.8%, respectively [8, 9].<br>MAE is one of the most common types of adverse events for hospital admitted patients, and the most frequent cause of preventable death [10]. 38% of MAE are serious or fatal, and 42% of those are preventable [10]. MAE has a significant impact on patients in terms of morbidity, mortality, adverse drug event, and increased length of hospital stay. In addition, it increases costs to clinicians and healthcare systems [11]. In the UK,26% of MAE were potentially serious, with fatal events which led patients to aspiration pneumonia and intracranial hemorrhage [12]. In Germany, 70% of all intravenous medications administered had at least one clinical error, and a quarter of these were serious errors likely to result in permanent harm to patients [13].<br>The most common factors which contribute to MAE are failure to check the patient's identification prior to administration, the storage of similar preparations in similar areas, and environmental factors like nurse interruptions whilst undertaking a drug round [3]. Other factors which contribute to MAE are characteristics of the nurse (age, sex, years of experience, year in the specific unit, nurse-to-patient ratio and educational status), route, and time of drug administration [14]. Inaccurate documentation and poor communication during change of shifts in the hospitals also contribute to MAE [15, 16].<br>Bar-coded medication administration (BCMA) and ensuring the patent identifications and following medication rights can help reduce medication administration error by 54–87% [6].Medication administration errors can also be prevented by voluntary reporting, direct observation, manual chart review, and computerized techniques [17]. Training and competency of the nurse, medication administration policies, continuous quality improvement efforts of the nurse, clear and accurate documentation, patient education, and teamwork help decrease medication administration error in hospitals [18].<br>Therefore, assessing the magnitude and associated factors of MAE will produce information that can be used by program managers and stakeholders in the planning and interventions of medication administration errors. Detecting the problem also helps to take corrective measurement for improving medication safety and quality, patient outcomes, adverse drug events, and quality of hospital services.
Objectives	3	***Specific objectives***<br>➢ To determine the magnitude of medication administration errors among nurse's at Felege Hiwot Referral Hospital in patient department.<br>➢ To identify factors associated with medication administration error.
**Methods**		
Study design	4	An institutional-based, cross-sectional study was conducted in Bahir Dar, Ethiopia, at Felege Hiwot Referral Hospital (FHRH).
**Setting**	5	The study was conducted in Bahir Dar town at Felege Hiwot Referral Hospital from March 24 - April 7, 2014. Bahir Dar is the capital city of Amhara region which is found 563 KM away from Addis Abeba. Felege Hiwot Hospital is one of the Referral Hospitals in Amhara region which is found at B/Dar city. The Hospital expected to provide service for more than five million people. FHRH has different departments and wards like medical (female and male), surgical, pediatric, gynecology, emergency, recovery, and ICU in which this research is focused on. In addition the Hospital has other departments like maternity, ophthalmology, OR, OPD, MCH rooms, and one private wing clinic and it has a total number of 135 diploma and 37 BSC nurses.<br><br>Data were collected and supervised by eight BSc. nurses and two MSc. nurses respectively. Before the actual work, data collectors and supervisors were given intensive training for two days about the objective of the study, the format of the questionnaire and checklist, procedures of observation and methods of reporting to supervisors and principal investigators. After the training, the

		questionnaire and checklist were pretested on five nurses who were working in Debre Markose Referral Hospital. Data on medication administration were collected through face-to-face interviews by using a structured questionnaire and by directly observing using checklist. In addition to this, immediately after observation, data on recorded observation were compared with the physicians order by referencing the patient's chart.
Participants	6	All nurses who were working at inpatient department from surgical, medical, pediatric, ICU, emergency, recovery and gynaecology ward during the study period were included.
Variables	7	Clearly define all outcomes, exposures, predictors, potential confounders, and effect modifiers. Give diagnostic criteria, if applicable<br>**Dependent/Outcome variable**<br>- Medication administration error<br>**Independent/Exposure variables**<br>➢ Socio-demographic characteristics of the nurse<br>o Age<br>o Educational levels (status)<br>o Year of working experience<br>➢ Nurse to patient ratio<br>➢ Shift of medication administration<br>o Night time, working time<br>➢ Route of medication administration<br>➢ Time of drug administration<br>➢ Interruption of the nurse during medication administration<br>o Like talking phone, other staffs, attendants, and patients<br>➢ Age of the patient
Data sources/ measurement	8*	Data on medication administration were collected through face-to-face interviews by using a structured questionnaire and by directly observing using checklist. In addition to this, immediately after observation, data on recorded observation were compared with the physicians order by referencing the patient's chart. The content of the data collection formats were design to record the nurse's demographics and work experience, patient's demographics and all data regarding the patient's medication intervention, date and time that specific drug was prescribed and administered, including the name of the drug, dosage, dosage given, frequency, and route of medication administration.
**Bias**	9	One of our efforts to address interobserver bias was by providing effective training as well as by retrain again and again before and during data collection. But for other kind of bias like Hawthorne effect we didn't do nothing since most of the time, MAEs occurred because of lack of knowledge rather than by lack of concentration, carelessness or being observed by someone else (as illustrated from an article entitled with "Validity and reliability of observational methods for studying MAEs".
Study size	10	Sampling was done using non-probability convenience technique, in which the sum of all the medications which were administered by 85 nurses was taken as the sample size. Therefore, all medication administration interventions to all patients in the inpatient departments by 85 nurses during the data collection period with different medication administration schedule were included.
Quantitative variables	11	First of all Bivariate analysis was used primarily to check which variables have association with the dependent variable. Then variables found to have p-value of less than or equal to 0.2 were entered in to Multiple Logistic regression for controlling the possible effect of confounders. Finally the variables which had statistically significant association were identified on the basis of OR with 95%CI.

Statistical methods	12	Bivariate analysis was used primarily to check which variables have association with the dependent variable. Then variables found to have p-value of less than or equal to 0.2 were entered in to Multiple Logistic regression for controlling the possible effect of confounders.
**Results**		
Participants	13*	(a) Out of 85 study samples, 82 nurses were interviewed and observed making the response rate of 96.5%.
		(b) The reason for non participation was they were not voluntary to be involved in the observational study.
Descriptive data	14*	(a) Out of 85 study samples, 82 nurses were interviewed and observed making the response rate of 96.5%. Majority (84%) of them were female. The mean age of the respondents was 31.13 years with SD of 6.4 years. Majority (85.4 %) of the respondents was diploma nurse and most (65.9%) of them had working experiences of less than 10 years.
		(b) There were no any missing data.
Outcome data	15*	From the total medication administration intervention, more than half (56.4%) were labelled as medication administration errors.
Main results	16	Variables which were significantly associated with medication administration error include nurses between the ages of 18-25 years [Adjusted Odds Ratio (AOR)=2.9, 95% CI (1.65,6.38)], 26-30 years [AOR=2.3, 95% CI (1.55, 7.26)] and 31-40 years [AOR=2.1, 95% CI (1.07, 4.12)], work experience of less than or equal to 10 years [AOR=1.7, 95% CI (1.33, 4.99)], nurse to patient ratio of 7-10 [AOR=1.6, 95% CI (1.44, 3.19)] and greater than 10 [AOR=1.5, 95% CI (1.38, 3.89)],interruption of the respondent at the time of medication administration [AOR=1.5, 95% CI (1.14, 3.21)],night shift of medication administration [AOR=3.1, 95% CI (1.38, 9.66)]and age of the patients with less than 18 years [AOR=2.3, 95% CI (1.17, 4.62)].
Other analyses	17	There were no any further analysis done like analyses of subgroups and interactions, and sensitivity analyses
**Discussion**		
Key results	18	- To determine the magnitude of medication administration errors: Out of 360 medication administration interventions, the majority (98.1%) of medications had at least one type of medication administration error. Among this, 42 (11.9%) had only one type medication administration error, 112(31.7%) had two type of errors, 168(47.6%) had three types of errors and the rest 31(8.8%) had more than three types of medication administration error. From the total medication administration intervention, more than half (56.4%) were labelled as medication administration errors. From 87 medication administration interventions which were given at medical unit, 42(47.2%) had medication administration error and also in emergency ward from 12 observed medications, all of it had medication administration error. Documentation error, technique error and wrong timing contributed for 315(87.5%), 263(73.1%), and 193(53.6%) of the medication administration errors, respectively.<br>- To identify factors associated with medication administration error: Based on the bivariate analysis, the factors found to be significantly associated with medication administration error were age of the respondent and the patient, nurse's working experience, interruption of the nurses during time of medication administration, shift of medication administration, nurse to patient ratio and route of medication administration.<br>Out of variables which were entered to multiple logistic regression, age of the respondents and the patient, nurse's working experience, interruption of the nurses at the time of medication administration, shift of medication administration, and nurse to patient ratio were found to be significantly

		associated with medication administration error at p-value of < = 0.05.
Limitations	19	Generalization of the findings presented should be made with caution because of the following limitations: small sample size and lack of random variation in a study's estimates were the main limitations of the study.
Interpretation	20	In conclusion, medication errors at the administration phase were highly prevalent in FHRH. Each medication and each patient had at least one type of medication administration error. Documentation error was the most dominant type of error followed by technique and time error respectively. Organizational factors such as error reporting systems and routine checks could possibly help in handling the problem of medication errors. Also, increasing nurse’s staffing levels, minimizing distraction and interruptions during medication administration by decreasing overcrowding and by using no interruptions zones and “No-Talk” signage are recommended to overcome MAEs. Hospital managers should strive to retain experienced nurses from leaving and train and supervise inexperienced nurses with a focus on medication safety. In addition, an administration control to provide convenient sleep hours will help nurses in improving sleep circadian rhythms and reduce MAEs.
Generalisability	21	Generalization of the findings presented should be made with caution because of the following limitations: small sample size and lack of random variation in a study's estimates were the main limitations of the study.
**Other information**		
Funding	22	Our heartfelt thank goes to University of Gondar for financial support.
