# Supplementary material for: Medication administration error: magnitude and associated factors among nurses in Ethiopia
Source: BMC Nurs. 2015 Oct 21;14:53. doi: 10.1186/s12912-015-0099-1 (PMC4618536; doi:10.1186/s12912-015-0099-1)
Supplement: Additional file 2: — English version questioner for Medication Administration Error: Magnitude and Associated factors among Nurses in Ethiopia. (PDF 305 kb) [file 12912_2015_99_MOESM2_ESM.pdf]

University of Gondar

College of Medicine and Health Sciences

Department of Nursing

A questionnaire and observational checklist prepared to assess magnitude and associated factors of medication administration error among nurses at Felege Hiwot Referral Hospital, Bahir Dar, Northwest Ethiopia, 2014.

**Part one: Face to Face interview questioner<br>1. Socio demographic characteristics of the nurses**		
**Sr No**	**Question**	**Response**
101	Age	------------------ in year
102	Sex	A. Male<br>B. Female
103	Religion	A. Orthodox<br>B. Muslim<br>C. Protestant<br>D. Catholic<br>E. Other (specify) _____________
104	Ethnic group	A. Amhara<br>B. Tigere<br>C. Oromo<br>D. Other (specify) _____________
105	Educational status	A. Diploma<br>B. Degree<br>C. Other (specify) _____________
106	Monthly salary	______________ in ETB
**2. Work related questions**		
201	Working experience of the nurse	_________________ in year
202	Working area	A. Medical ward<br>B. Gynecology ward<br>C. Pediatric ward<br>D. Surgical ward<br>E. ICU<br>F. Emergency<br>G. Recovery
203	Duration in the specific/present unit	---------------- in month
204	Nurse to patient ratio	1 to -----------------------

Part two: Filled by direct observation of the nurse during medication administration

Instruction: Please observe the nurse starting from medication preparation to administration and write the exact information of the medication (any errors) based on the medication administration checklist. Use one format for a single patient medication administration process

Sr. No	Question	Response
301	Patient 's age	________________ in year
302	Patient's sex	A. Male<br>B. Female
303	Time in which medication administered (exact time of medication administration)	
304	Shift of medication administration	A. Day time<br>B. Night time
305	Is there any interruption (like phone, talking with other staffs or clients) of the nurse at the time of medication preparation up to administrations	A. Yes<br>B. No

306. A table used to fill the type of the drug given to the patient by the observed nurse and the type of medication administration errors

Type of error								
**Name of the drug**	**Route of administration**	**Unauthorized drug error**	**Time error**	**Wrong route**	**Missed drug error**	**Dose error**	**Technique error**	**Documentation error**
Ampcline								
Cry. Penciline								
Gentamicine								
Lasix								
Chloroamphnicol								
Ceftraxione								
Diclofinace								
Tramadole								
Quinine								
Chlorpromazine								
Diazepam								
Insulin								
Cephazdine								
Vancomycin								
Others								

307. Examples on type of medication administration error observed at each observed medications……………………………...

Part three: Competency checklist for medication administration

Section 1: Core competencies check lists to assess technique errors of medication administration

No	Check list	Yes	No
1	check expiration date		
2	Compare medication with order		
3	check label:		
	a. When taking from shelf		
	b. Before withdrawing/pouring		
	c. Before returning to shelf		
**a**	**For Intramuscular Injections:**	**Yes**	**No**
	Identifies the right patient		
	Explain the procedure		
	Ask about medication allergies		
	Ask if patient faints with injections		
	Select appropriate site		
	Cleanse site appropriately		
	Wearing gloves		
	Stretches skin taut		
	Injects at 90 degree angle		
	Aspirates		
	Injects appropriately		
	Withdraw the syringe appropriately		
**b**	**For Subcutaneous Injections**	**Yes**	**No**
	Identify the right patient		
	Explain the procedure		
	Ask about medication allergies		
	Ask if patient faints with injections		
	Select appropriate site		
	Cleanse site appropriately		

No	Check list	Yes	No
	Wearing gloves		
	Skin grasped		
	Inject at 45 degree angle		
	Aspirates		
	Follows appropriate protocol if blood aspirates		
	Injects appropriately		
	Withdraw appropriately		
	Observes patient for reaction		
**c**	**Intravenous Injections:**	**Yes**	**No**
	Identify the right patient		
	Explain the procedure		
	Ask about medication allergies		
	Check for compatibility of medication and IV fluid		
	Provide information on the medication		
	Select appropriate site or observe the canula site		
	Wearing gloves		
	Inject appropriately		
	Withdraw appropriately		
	Proper syringe disposal		
	Observe the patient for reaction		
**d**	**Oral medication (if patient is self medicated)**	**Yes**	**No**
	Put the patient medication in locked cabinet		
	Clarify to the patient about the exact dose		
	About the exact time		
	Observe the patient at the time of swallowing		
	Observe the patient for reaction		

Section 2: Common antibiotic and the appropriate standard amount of diluents to be added for IV injections and its rate of administrations (Used for assessing technique error)

No	Name of antibiotic	Preparation	Amount of diluents for Iv injection	Rate of administration time
1	Ceftriaxone	250 mg	2.4ml	Slowly 6-7 minutes
		500 mg	4.8ml	
		1 gm	9.6ml	
		2 gm	19.2ml	
2	Cloxacilin	250 mg	3-5ml	Slowly 3-4 minutes
		500 mg	3-5ml	
3	Chloroamphnical	500 mg	5ml	Within 1 min
		1 gm	10ml	
4	Cry. pencline	1 MIU	2ml	Slowly 3 minutes
5	Ampcline	250 mg	1ml	Slowly 3-5 minutes
		500 mg	1.8ml	
		1 gm	3.5ml	Slowly 5 minutes
6	Vancomicine	500 mg	100ml N/S	30 minutes
		1 gm	200ml N/S	60 minutes
7	Cephaziden	1 gm	10ml	Slowly 3 minutes

Section 3: Check list which describes each of medication administration errors

No	Description	Yes	No	Type of error
1	Fail to administer a prescribed medication while the drug available at the patient bed side			Missed drug error
2	Administered was not authorized by the prescriber which is different from the physician order			Unauthorized drug error
3	Perform less than 50% among the procedure put at the technique competency checklist for Medication Administration			Technique error
4	Medication dose or quantity different from the prescribed			Wrong dose error
5	There is thirty min difference between the ordered time (exact administration time) and the time the medication is administered			Wrong time error
6	The medication administered through different routes other than the physician orders			Wrong route error
7	The medication which was administered to the patient not documented in medication administration record sheet			Documentation error

Thank you!!!!
